# A Cluster-Randomized Controlled Intervention Study to Assess the Effect of a Contact Intervention in Reducing Leprosy-Related Stigma in Indonesia

**DOI:** 10.1371/journal.pntd.0004003

**Published:** 2015-10-20

**Authors:** Ruth M. H. Peters, Dadun, Marjolein B. M. Zweekhorst, Joske F. G. Bunders, Irwanto, Wim H. van Brakel

**Affiliations:** 1 Athena Institute, Faculty of Earth and Life Sciences, VU University, Amsterdam, The Netherlands; 2 Faculty of Public Health, Universitas Indonesia, Depok, Indonesia; 3 Athena Institute, Faculty of Earth and Life Sciences, VU University, Amsterdam, The Netherlands; 4 Athena Institute, Faculty of Earth and Life Sciences, VU University, Amsterdam, The Netherlands; 5 Centre for Disability Studies, Faculty of Social and Political Sciences, Universitas Indonesia, Depok, Indonesia; 6 Netherlands Leprosy Relief, Amsterdam, The Netherlands; Komofo Anokye Teaching Hospital, GHANA

## Abstract

**Background:**

Can deliberate interaction between the public and persons affected by leprosy reduce stigmatization? The study described in this paper hypothesises that it can and assesses the effectiveness of a ‘contact intervention’.

**Methods/Principal Findings:**

This cluster-randomized controlled intervention study is part of the Stigma Assessment and Reduction of Impact (SARI) project conducted in Cirebon District, Indonesia. Testimonies, participatory videos and comics given or made by people affected by leprosy were used as methods to facilitate a dialogue during so-called ‘contact events’. A mix of seven quantitative and qualitative methods, including two scales to assess aspects of stigma named the SDS and EMIC-CSS, were used to establish a baseline regarding stigma and knowledge of leprosy, monitor the implementation and assess the impact of the contact events. The study sample were community members selected using different sampling methods. The baseline shows a lack of knowledge about leprosy, a high level of stigma and contrasting examples of support. In total, 91 contact events were organised in 62 villages, directly reaching 4,443 community members (mean 49 per event). The interview data showed that knowledge about leprosy increased and that negative attitudes reduced. The adjusted mean total score of the EMIC-CSS reduced by 4.95 points among respondents who had attended a contact event (n = 58; p <0.001, effect size = 0.75) compared to the score at baseline (n = 213); for the SDS this was 3.56 (p <0.001, effect size = 0.81). About 75% of those attending a contact event said they shared the information with others (median 10 persons).

**Conclusions/Significance:**

The contact intervention was effective in increasing knowledge and improving public attitudes regarding leprosy. It is relatively easy to replicate elsewhere and does not require expensive technology. More research is needed to improve scalability. The effectiveness of a contact intervention to reduce stigma against other neglected tropical diseases and conditions should be evaluated.

## Author Summary

Stigma plays an important role in several neglected tropical diseases such as leprosy, Buruli ulcer, lymphatic filariasis, onchocerciasis and leishmaniasis. It negatively impacts individuals affected, and often also their families, and even communities. There are different ways to address stigma. One promising intervention is called ‘contact’. The principle is that personal contact between persons affected by a stigmatized condition and the public will demystify incorrect information, break the stereotypes and generate empathy. This in turn is believed to reduce stigma. In this paper the authors report a study that investigated the effect of a contact intervention that aimed to reduce leprosy-related public stigma in Cirebon District, Indonesia. During this intervention 91 so called ‘contact events’ were organized at a local level, for instance, in schools, villages, halls and mosques. The results show that through education, testimonies (direct contact), videos and comics made by people affected by leprosy (in-direct contact) knowledge about leprosy increased and personal attitudes improved substantially.

## Introduction

*Interviewer*: *Who would be able to make the community accept people affected by leprosy*? *Community member*: *Encouragement from the neighbours might help the affected people*. *If they stay at home all the time*, *they might feel bored*. *After getting encouragement*, *they might get back to community life like usual*. *(woman*, *not affected by leprosy*, *48 years)*

We were positively surprised by some of the answers given by community members in Cirebon District, Indonesia to questions like the one above. Several community members said that they themselves—as members of a community—and the community as a whole, play a major role in dealing with leprosy and, in particular, in the stigma, discrimination and social exclusion many people affected by leprosy experience. The questions asked made community members reflect on their own role and, for instance, one community member said: *“I will start by changing myself”*. Another community member said he and other key persons should give information about leprosy to the community, but underlined that his current lack of knowledge prevented him from giving information that would *“satisfy”* the people. These examples illustrate the willingness of some people in this particular community to bring about a change, but also the importance of effective interventions to facilitate this change.

Stigma plays an important role in field of mental health, HIV/AIDS and several neglected tropical diseases (NTDs) such as leprosy, Buruli ulcer, lymphatic filariasis, onchocerciasis, leishmaniasis and Chagas disease [1–6]. It affects the lives of individuals affected, and often their families, and even communities. In addition, it is also a barrier to effective management and control of these diseases [3]. Leprosy is an archetype of stigmatised health conditions. In 2013, 215,656 new leprosy cases were reported worldwide [7]. Indonesia—with 16,856 new cases in 2013 –ranks third after India and Brazil on the list of countries with the highest number of recorded new cases [7]. The negative consequences of leprosy-related stigma have been studied in different countries across the globe [8–13].

Several types of interventions to reduce leprosy-related stigma have been developed and tried such as counselling, self-help groups, advocacy and socio-economic development [14–20]. So far, no attempts have been made to systematically evaluate their effect. In this paper, we will focus on one of the most promising interventions, particularly if combined with education, to address leprosy-related stigma at the community level: the contact intervention [11,15]. Most experience with the contact intervention has been acquired in the field of mental health but there are also examples in the field of HIV/AIDS [4,15,18,21–26]. According to Brown et al., contact refers to “an environment in which the general population can interact with the stigmatized group” [15]. Contact can be either direct (e.g., face-to-face conversation or testimony) or indirect (e.g., through media or recorded video). The rationale—which originally comes from the field of social psychology—is that a more personal relationship through direct or indirect contact will demystify and discharge incorrect information, invalidate stereotypes and generate empathy, which in turn reduces stigma and prejudice [15,27–29]. Dalal describes that “attitudes develop and change whenever we are exposed to a new experience and information” and that, once formed, “these attitudes become the basis for shaping our new experiences” [29]. He highlights the importance of strong an compelling discrepant information “to create some dissonance in the mind of the person” [29]. Also Howarth argues that it is possible to “unsettle, challenge and potentially transform representations and practices that stigmatize” through a collective enterprise; coming together in dialogue, debate and critique [30]. She underlines the importance of seeing stigmatized individuals as agents instead of objects or victims of stigma.

There have been some critical notes as well. We will highlight three. First, not all contact is effective. Corrigan & Shapiro [19] reflect on mental health care providers who “have a great deal of contact with individuals with mental illnesses, yet they are among the most stigmatizing”. In addition, Kunda & Oleson’s work on stereotypes questions the effectiveness of this approach, suggesting that people who encounter an individual that is “deviant” will question the accuracy of their own stereotypes, but nevertheless tend to regard this individual as an exception to the stereotyped group [31]. Second, putting an emphasis on stigma through interventions could re-enforce or amplify stigma. Howarth noted: “as stigma develops from and maintains relations of inequality, attempts to challenge stigma will be highly contested” [30]. Caution and care is thus needed, since the persons involved may be asked to disclose private information, which can have negative consequences [14]. Finally, the effectiveness of the interventions remains largely unknown, because testing of the effectiveness of stigma-reduction interventions is not easy to do [14,32].

Hence, it is important to develop a contact intervention that facilitates effective interaction, empowers the persons affected by leprosy and engages them as agents of change. This is what the Stigma Assessment and Reduction of Impact (SARI) Project in Indonesia, of which this study is a part, intended to do. In this paper, we assess the effectiveness of a contact intervention using education, testimonies, participatory videos and comics in reducing leprosy-related public stigma in Cirebon District, Indonesia. Based on the insight from this study, we give recommendations for health policy and further research regarding stigma reduction.

### Theoretical framework: Public stigma

The body of theory and research on stigma has been developed over fifty years since its original conceptualization by Goffman [33]. Over time, new conceptualizations of health-related stigma have been developed to improve the relevance of the concept, by scholars from different scientific disciplines (e.g. psychology, psychiatry, public health, sociology, anthropology) and sometimes for specific health conditions [34–38]. Not surprisingly, these conceptualizations of stigma vary [34] and a major critique is that the concept of stigma has not become any clearer. Recently, Staples argued that stigma “can become a lazy shortcut for multiple ‘social aspects’ of leprosy” [39]. He underlines the importance of a more reflexive approach in the light of the critiques.

For this paper it is important to distinguish between the interacting categories or levels of stigma that are articulated in the literature. Weiss [3] identified two categories (stigmatized and stigmatizers), Bos et al. [38] three levels (individual, social and structural) and Heijnders & van der Meij [14] five levels (intrapersonal, interpersonal, community, institutional and structural). The contact intervention primarily aims to reduce stigma from so-called ‘stigmatizers’ at the social or community level.

Link & Phelan’s work on stigma is interesting in this context, as it gives more in-depth insights in the processes operating at this level. In their definition, stigma exists when “elements of labelling, stereotyping, separating, status loss and discrimination co-occur in a power situation that allows these processes to unfold”. The following five components need to converge:

In the first component, people distinguish and label human differences. In the second, dominant cultural beliefs link labelled persons to undesirable characteristics—to negative stereotypes. In the third, labelled persons are placed in distinct categories so as to accomplish some degree of separation of “us” from “them.” In the fourth, labelled persons experience status loss and discrimination that lead to unequal outcomes. Finally, stigmatization is entirely contingent on access to social, economic, and political power that allows the identification of differentness, the construction of stereotypes, the separation of labelled persons into distinct categories, and the full execution of disapproval, rejection, exclusion, and discrimination [24].

Link & Phelan’s work focuses on the nature and consequences of stigma rather than the sources. These are also important for this study. Sermrittirong & van Brakel categorized the causes of leprosy-related stigma in external manifestations of the disease, cultural and religious beliefs, fear of transmission, association with people considered inferior and public health-related interventions [40]. In conclusion, this paper focuses on the impact of a contact-based intervention on public stigma, targeting the processes of labelling, stereotyping and separation. The interventions incorporate the underlying causes of stigma by addressing the knowledge, beliefs, fears and questions people have regarding the stigmatised condition.

### The contact intervention of the SARI project

The contact intervention was developed over the course of the first one and a half years of the SARI project. The SARI project was carried out by a research team including ten research assistants (RAs) from around Cirebon who spoke the local languages and researchers from VU University Amsterdam and Universitas Indonesia. Three RAs had either a physical or visual disability and one was affected by leprosy. One of the PhD students and the principal investigator (I) are also persons with a disabilities. Conditions under which the contact intervention seems to have the greatest impact articulated in the literature were considered during the design of the intervention. These include an equal status between participants, one-on-one contact, frequent contact, contact with individuals who mildly deviate from the stereotype, an element of education, high levels of intimacy, real-world opportunities to interact and institutional support [14,19]. In addition, the SARI teams’ experience and understanding of the local context (see also [8,9,41]) at that time were important considerations for the development of the interventions. We decided to organise so called ‘contact events’ at a local level, for instance, in schools, village halls and mosques. The local RAs together with persons affected by leprosy became responsible for the organisation of the events and had to make sure the events fitted into and made optimal use of the local social structures and context. Each contact event had two core elements.

The first core element is contact between affected persons and the public. An exploratory study was conducted in Cirebon, which resulted in a range of possible methods to create direct and indirect contact. In the end, testimonies were chosen as the direct method and participatory videos and comics made by people affected by leprosy as the in-direct method. The rationale for this choice was: (i) participants of the exploratory study thought these methods would be effective, (ii) the stories and messages would come from people affected by leprosy themselves, (iii) the testimonies and the development of participatory videos and comics on their own were expected to be valuable and empowering experiences for the participants, (iv) the development process would be relatively inexpensive, which is important for scalability, (v) material was expected to be attractive, distributable and easy to understand, and (vi) the SARI team could build upon previous knowledge and experience from organisations like InsightShare (see http://www.insightshare.org/) and World Comics (see http://www.worldcomics.fi/).

During the course of the project, two participatory videos were developed telling stories that the makers wanted to tell to the community. They are titled ‘*Pastikan badai sirna*’ (Surely the storm has vanished) and ‘*Empat sahabat yang selalu berbagi*’ (Four friends who always share) (paper in progress). In addition, 32 comics (4 panels each) in black and white were developed by young people affected by leprosy depicting their life experiences and again bringing across a message they wanted to share with the community. Not every contact event incorporated all three methods; a selection was made based on the available time, venue and interest of the audience.

The second core element is education. From the exploratory study we concluded that there are misconceptions about the causes of leprosy and the mode of transmission, and a lack of understanding regarding the social consequences of leprosy in Cirebon District (see also Peters et al. [8]). In our opinion, the common view that stigma is caused by a lack of knowledge and that “education is therefore a panacea for stigma” [32] is wrong. This opinion is supported by others authors [5,15,39,42]. But that is not to say that education is not an essential component [15]. Wong states the importance of talking about local beliefs and changing these gradually through exploration and clarification [42]. The messages spread during the contact events using an interactive presentation are displayed in Box 1. During the event the audience was encouraged to share the information they gained with family members, friends and others.

**Box 1. Key Messages Highlighted during the Contact Events of the SARI Project**

- Leprosy is caused by a bacterium and not *guna guna* (witchcraft/ black magic) nor *turun temurun* (hereditary)
- Leprosy can spread between people who have extensive and close contact
- Only a very small percentage of the population is susceptible to develop leprosy
- The symptoms of leprosy are skin lesions
- Diagnosis can be provided at the *puskesmas* (Community Health Centres (HC))
- Free medication is available at the HC
- Treatment with medicine prevents the spread of the disease
- Leprosy is curable
- Impairments on hands and feet can develop when treatment is not started on time
- We should not stigmatize people affected by leprosy, but instead support them
- People with leprosy have the same rights as other people

## Methods

The study area is Cirebon District, located in West Java. It has a relatively high number of new cases annually and—according to national experts—a high level of leprosy-related stigma in comparison to other districts and no initiatives to address this. Administratively, Cirebon District consists of 40 *kecamatan* (sub-districts), 412 *desa* and 12 *kelurahan* (both administrative villages) [43]. The study used a cluster-randomized controlled intervention design, which was preceded by an exploratory study. The intervention study and exploratory study were carried out in different sub-districts. The 30 sub-districts not involved in the exploratory study were randomly allocated a paired intervention or became control area: (i) ‘Counselling—Contact’, (ii) ‘Contact—Socio Economic Development (SED)’, (iii) ‘SED—Counselling’, and (iv) ‘Control’. The SARI project ran from 2010 till 2015. The baseline study was executed in 2011. The contact events were organised between November 2012 and December 2013. The final survey was conducted in 2014. Respondents that attended a contact event are referring to an event that had taken place between 3 and 20 months before the final interview.

### Mixed methods

A variety of both qualitative and quantitative research methods have been applied to assess stigma in community members in Cirebon District (see Table 1 for an overview). Each will be explained in more depth.

**Table 1:** Overview methods.

Phase	Contact
Baseline	EMIC—CSS*
	SDS**
	In-depth interviews
Continuous monitoring and evaluation	6-QQ*** before, after and after >3 months
	Short informal interviews after contact event
	Reports
Final survey	EMIC—CSS
	SDS
	Additional questions
	FGDs**** after >3 months

* Explanatory Model Interview Catalogue Community Stigma Scale [44,45]

** Social Distance Scale [44,46]

*** 6-Question Questionnaire

**** Focus Group Discussions

#### Two scales: EMIC-CSS and SDS

Two scales were selected to assess public stigma: the Explanatory Model Interview Catalogue Community Stigma Scale (EMIC-CSS) and the Social Distance Scale (SDS). According to current international standards, both instruments have adequate cultural validity to assess public stigma in leprosy in the Bahasa Indonesia-speaking population of Cirebon District [44]. The EMIC-CSS assesses the views of the community in general towards leprosy. The scale has 14 items and covers aspects of life that are often affected by stigma or coping strategies, such as concealment, marriage (prospects) and work. The scale has four response options; yes (2 points), maybe (1), no (0) and do not know (0). In contrast, the SDS assesses the personal views of the respondent and started with reading out a vignette describing a male named Rahman (for male respondents) or female named Rahmi (for female respondents) affected by leprosy. The scale has seven items representing different degrees of social distance. For example, the respondents’ willingness of having someone like Rahman or Rahmi as a tenant in their house or as a caretaker of their children. The items have four response options; definitely willing (0 points), maybe willing (1), maybe not willing (2) and definitely not willing (3). The scales are preceded by several questions on demographics. In the final survey several questions were added for respondents who had attended a contact event, such as “What do you remember from the event?”, “Did you share this information to others? and, if yes, “To how many?”

The selection of baseline and final survey respondents was done as follows; people affected by leprosy were invited to different HCs for an interview (data are not part of this study) and at each HC, three persons affected by leprosy were randomly selected (using papers with numbers). Their *Rukun Tetangga* (RT, smallest administrative level with approximately 10–20 households) was visited by two or three RAs of the SARI project. The RAs first visited the head of the RT to introduce themselves and explain the purpose of the SARI project. Using convenience sampling, they then selected three community members from this or a neighbouring RT. Two were key persons. A key person is defined as a person who can reach a larger group of the community members and whose knowledge and reputation could influence their attitude. Key persons included head RT, head *Rukun Warga* (several RTs), head of village, village office staff, teacher, health service volunteer or religious leader. The third person, was a general community member about the same age and sex of the person affected interviewed that morning. To achieve adequate power for comparison between the baseline and final surveys, we calculate a required sample size of at least 200 community members (at least 50 per intervention area).

#### In-depth interviews

From all EMIC-CSS and SDS baseline respondents, 60 (15 per intervention area) were selected for an in-depth interview at their own house, using purposive sampling to ensure adequate representation of sexes, ages and roles in the community. The topics addressed were: (i) general information about the disease leprosy (ii), situation of persons affected by leprosy, (iii) own experience with persons affected by leprosy, (iv) public opinion towards leprosy, and finally, (v) role and responsibility of others actors.

#### 6-question questionnaire

A 6-Question Questionnaire (6-QQ) was developed by the SARI team as a simple and rapid tool to get a quick impression of the change in knowledge (2 questions) and attitudes of participants (4 questions). Questions included, “Can leprosy be cured?” and “Do you feel comfortable when you meet a person affected by leprosy?” The answer options were ‘yes’ or ‘no’. In addition, age, sex, job and status of the respondents within the community were collected. The questionnaire was piloted and some questions were adapted before implementation. Before and after the contact events, participants were asked to fill in the 6-QQ. The participants were selected using convenience sampling. At least three months after the contact event took place, several participants were contacted by telephone or visited to answer the 6-QQ again. This will be referred to as post-3-month data.

#### Short informal interviews

To get a quick impression of the perspectives of the audience on the contact event, short informal interviews were conducted right after the events. People in the audience were purposively selected, based on characteristics such as age, sex and role in the community. The main questions asked during these interviews were: “What is your opinion about the event?”, “What were you views on leprosy before the event and why?” and “What are your views on leprosy after the event and why?”

#### Reports

The RAs wrote a report about each contact event. This report included general information (e.g. location, type of audience), observations and some pictures.

#### Focus group discussions

Three FGDs were held with participants who attended a contact event at least three months earlier. Participants were students, village leaders and women who knew each other from religious gatherings. Themes addressed during the FGDs were (i) the recollection of the contact event, (ii) any changes experienced due to the contact event and, (iii) the disclosure of information to others.

### Data analyses

The interviews and FGDs were recorded, transcribed and translated to English together with the summaries of informal interviews. The quantitative data was entered in an Epi Info for Windows database (version 3.5.3) and analysed using Stata 12.1 and SPSS 21. The qualitative data was analysed using N-Vivo and WeftQDA. Maps were made with QGIS 2.1.0. Demographic variables included sex, age (in years), marital status (yes/no), education, profession, household income per day (in Indonesian Rupiah (IDR)), key person (yes/no) and knowing a person affected by leprosy (yes/no). The respondents were asked for either income per day or income per month; the latter was converted into one variable ‘household income per day’ by dividing the income per month by 30.5. The differences between baseline and final survey respondents were tested using a Chi-square test for categorical variables and t-test for continuous variables. To investigate the effect of the interventions we calculated means and performed univariate and multivariate regression analyses. We used a backward elimination procedure considering the P-values and the model fit (R^2^). P values less than .05 were taken as significant. In addition, the effect size (ES) was calculated, which is the difference between the mean (M) total score pre (1) and post (2) intervention divided by the pooled standard deviation (SD) [47]. The formula is:

$$ES\;=\;\frac{M1-M2}{\sqrt{\left({(SD1}^{2}+SD2^{2})/2\right)}}$$

Following Cohen, an ES of 0.2 is considered as small, 0.5 as moderate, and 0.8 as large [47]. For the maps, the contact event in villages with 1 contact event are visualised at the centroid of the administrative boundary of the village. The contact events in villages with more than 1 contact event are visualised with the random point generator available in QGIS.

### Ethical considerations

The study was approved by the Ethics Committee of Atma Jaya University, the Sub-Directorate for Leprosy and Yaws, Ministry of Health, the Provincial Public Health Office, West Java, and the District Health Office, Cirebon District. Written informed consent was obtained from the participants. The control area in this study was a “care-as-usual” area. No incentives were offered to interviewees other than a small token of appreciation, such as a drinking mug or t-shirt, in particular when participants were interviewed more than once.

## Results

### Socio-demographics study subjects

#### EMIC-CSS, SDS and additional questions

During the baseline 262 and during the final survey 402 respondents were interviewed. Of these observations, respectively 20 (7.6%) and 3 (0.7%) were omitted due to missing values and 29 (11.1%) and 24 (6.0%) were omitted due to a language used other than Bahasa Indonesia. This left 213 observations in the baseline and 375 in the final survey. The socio-demographic characteristics of the respondents are shown in Table 2. Significant differences between respondents in the baseline and final survey were found for sex, age, profession, household income and being a key person or not. Of the 375 respondents in the final survey, 58 respondents attended a contact event.

**Table 2:** Socio-demographic characteristics of the quantitative baseline and final survey subjects.

Variables	Baseline (n = 213)*	Final survey (n = 375)*	P-value **
Sex			
Female	131 (61.5)	185 (49.3)	0.004
Age (in years)	42.2 (12.4)	40.3 (10.5)	0.045
Marital status			
Married	194 (91.1)	333 (88.8)	0.384
Education			
No education	9 (4.2)	22 (5.9)	0.686
Primary/secondary school	74 (34.7)	129 (34.5)	
High school or university	130 (61.0)	223 (59.6)	
Profession			
Paid job	102 (48.1)	168 (45.2)	<0.001
Own business or farmer	68 (32.1)	98 (26.3)	
Housewife	20 (9.4)	84 (22.6)	
Other	22 (10.4)	22 (5.9)	
Household income per day (IDR)	43,484 (38,960)	55,338 (74,264)	0.038
Key person			
Yes	138 (67.0)	188 (51.1)	<0.001
Know person affected by leprosy			
Yes	158 (77.1)	269 (73.3)	0.319

* Values given as count + (column percentage) or mean + (SD)

** Overall group differences, based on t-test for continuous variables and X^2^ statistics for categorical variables.

#### In-depth interviews

In total, 49 in-depth interviews were conducted with community members during the baseline. Of the 49 interviewees, 19 (39%) were women and the average age of the interviewees was 40.5 (youngest 20 and eldest 65).

#### 6-QQ

The questionnaire was filled in by 769 participants before and after the contact events. In total, 114 participants were contacted at least three months after the contact event to fill in the 6-QQ again. The pre and post dataset had 44.6% men and 55.4% women, with a mean age of 35.5 years. The post-3-month group comprised 28.1% men and 71.9% women with a mean age of 33.7 years.

#### Short informal interviews

In total, 89 short informal interviews took place after the contact events. Of these interviewees 40.4% were men and 59.6% women and the average age was 38.1 years.

#### Focus group discussions

Twenty-eight community members participated in the FGDs: village leaders were all men (N = 10), women of religious gatherings (N = 6) and a mixed group of both male and female students (N = 12).

### Baseline

#### Knowledge

One of the first questions asked in the in-depth interview was “What is leprosy?” A diversity of responses was the result ranging from the frequent “I don’t know” to more neutral or factual responses as leprosy is a “skin disease” and “contagious disease”. In addition, some responses were value laden, for example, leprosy is “worrisome”, “horrible”, “disgusting”, “extremely grievous” and “a curse”. In contrast, there were also responses underlining that leprosy is not frightening or just a normal disease. Most community members knew that leprosy is a contagious disease. Some interviewees said leprosy spreads easily, while others thought that the “chance is small” or “it takes a long time to spread”. There was ambiguity on how the disease spreads. Community members said that they or the community in general believes that leprosy spreads through: physical contact such as shaking hands, saliva, consuming the food or drinks prepared by a person by leprosy, using the same objects such as table ware, towels or clothes, genetics, mosquito bite and sexual intercourse. A few said that leprosy is not contagious once someone is on treatment or is cured. Several mentioned the importance of a healthy life style in preventing or having a lower risk of getting leprosy. Almost all respondents considered leprosy to be curable. Several interviewees were very sure, but others less so. Some community members noted that it is not easy to cure leprosy and that it will take a long time. Some differentiate between newly infected persons who can get cured and to those that have the disease for a while.

#### Labelling, stereotyping, separation, status loss and discrimination

Community members expressed that it varies how people affected are treated in their communities. Sometimes people affected by leprosy are accepted by the community but at others times they are not. Interviewees gave examples of communities were people affected are not accepted. They said that the community members feel “uncomfortable”, “scared”, “disgusted” and “terrified”. But more important is that processes as labelling, stereotyping, separation, status loss and discrimination are widespread as illustrated in Table 3. An illustrative quote:

*Interviewee*: *If he is badly affected and people know that he is affected by leprosy*, *he will not have any friend*.

*Interviewer*: *What if he is not so badly affected*?

*Interviewee*: *Same*. *There was a case like that here…*. *For example if we want to do a visit*, *someone will say*, *“Do not go there or you will get infected*.*” Something like that… Yes*. *And the family of the affected may say*, *“Don’t shame yourself*. *Get in the house*.*” (woman*, *37 years)*

**Table 3:** Examples of stigma and inclusion from respondents themselves or community members.

Examples of labelling, stereotyping, separation, status loss and discrimination	Examples of inclusion, compassion, care and support
have “some prejudice about the person”	“mind their own business”
“generalize”	treat people affected “as usual”
“look down on the person”	see them “part of the community”
think about the affected as “bad people”	see them as “one of our people”
believe they “deserve the disease”	“feel sympathy”
“keep a distance from the affected”	feel “sorry for them
“are reluctant to shop at [their] place”,	“approach them”
do “not want to buy snacks from [them]”,	“advise”
“isolate the affected person”	“support them to seek treatment”
“send them a few meters away”	“pray for [their] health”
“mock [them]”	“give moral support”
“forbid [them] to apply for any job”	“keep close and embrace them”
	“help who is in difficulty”
	“pay for the medication”
	“take them sailing”

Whether a person is on treatment or cured can change the response from the community, but sometimes it does not. The most important reason given for not accepting people affected is the fear of getting infected, but a few times also disgust, the smell of wounds and fear of impairment were mentioned. The importance of knowledge is highlighted in this quote, but also what interviewees refer to as the “heart”:

*Well*, *since I have a lack of knowledge*, *until this day*, *I always avoided them because I am afraid of getting infected*. *It is normal*, *isn’t it*? *I don’t want my legs to be cut*. *[later in the interview] Well*, *every person has a different heart and a different way of thinking*, *some [community members] treat [people affected by leprosy] the same*, *while others treat them differently*. *If it’s me*, *I think I will treat them differently*. *(woman*, *53 years)*

Other, perhaps, more underlying reasons also came up in the interviews. These are the stories or warnings told by their parents or elderly people in the community. An interviewee said that the problem is that people believe the stories and spread this information to others.

*People who are affected by leprosy should be banished to the forest [laughs]*. *They should not live together with other people in the village*. *Their houses should also be burnt*. *That was what my parents told me*. *(man*, *54 years)*

*They might say something like*, *“Be nice*, *or you will get leprosy*!*” to bad children who like to rebel against their parents*. *(woman*, *21 years)*

A few interviewees were aware of the impact exclusion might have on the persons affected and acknowledge that people affected by leprosy face a double burden: the condition and the attitudes of community members:

*People affected by leprosy definitely feel sad to have leprosy*. *Besides having leprosy*, *they have to accept that the people avoid being near them*. *(man*, *33 years)*

Equally important are interviewees that stated the exact opposite and depict care, support, compassion and inclusion (see Table 3). A few respondents talked about the rights and dignity of the people affected by leprosy. Two quotes to illustrate this more profoundly:

*In my opinion*, *isolating an affected person is not human*. *(man*, *52 years)*

*Why should they be excluded*? *Leprosy is just a normal disease*…. *Whatever the case is*, *they have their own right to communicate and socialize with other people*, *so if someone says that people affected by leprosy need to be excluded*, *I am against that*. *(man*, *38 years)*

### Contact events

**Fig 1:**
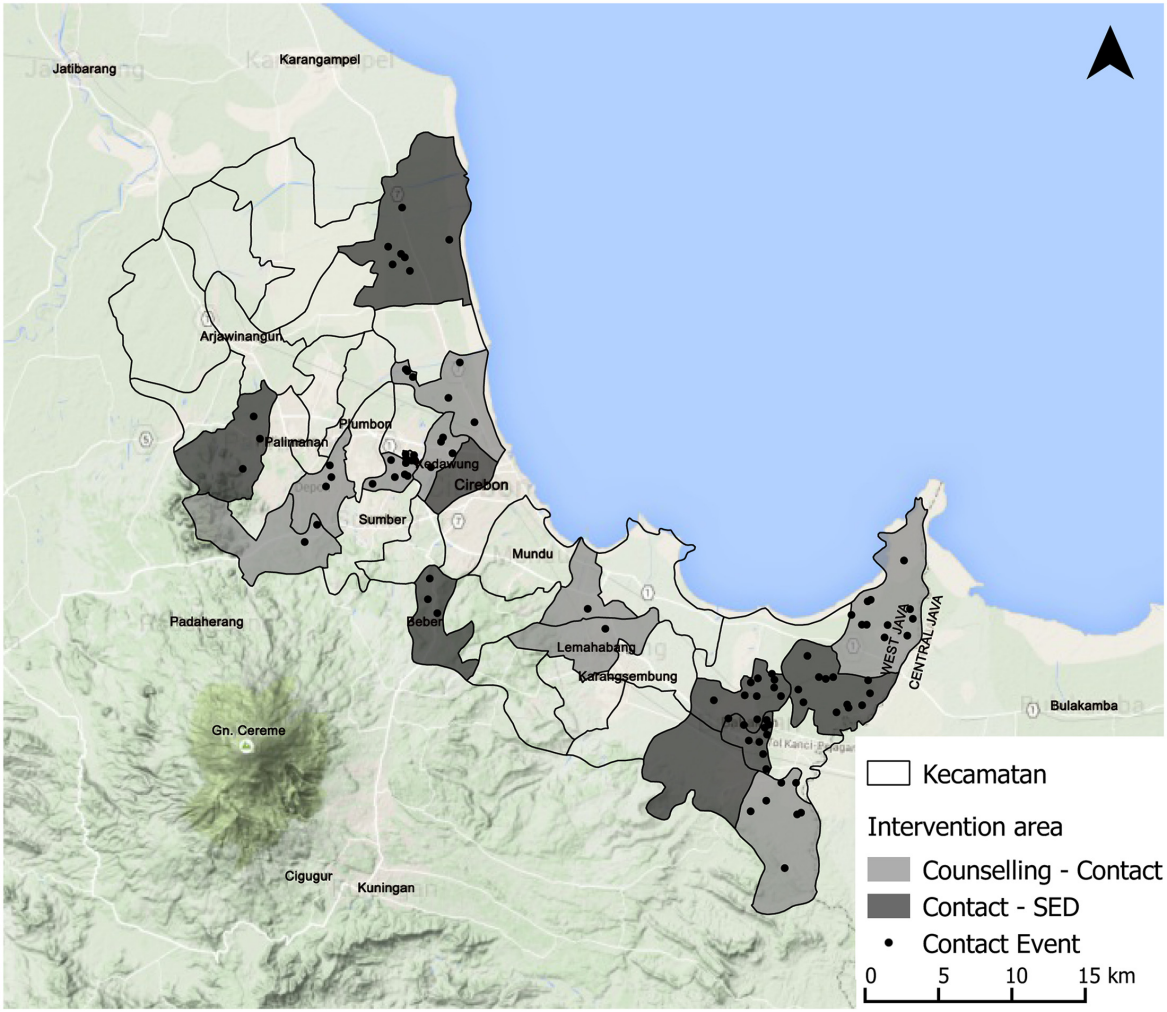
Contact intervention areas in Cirebon District and location contact events.

**Fig 2:**
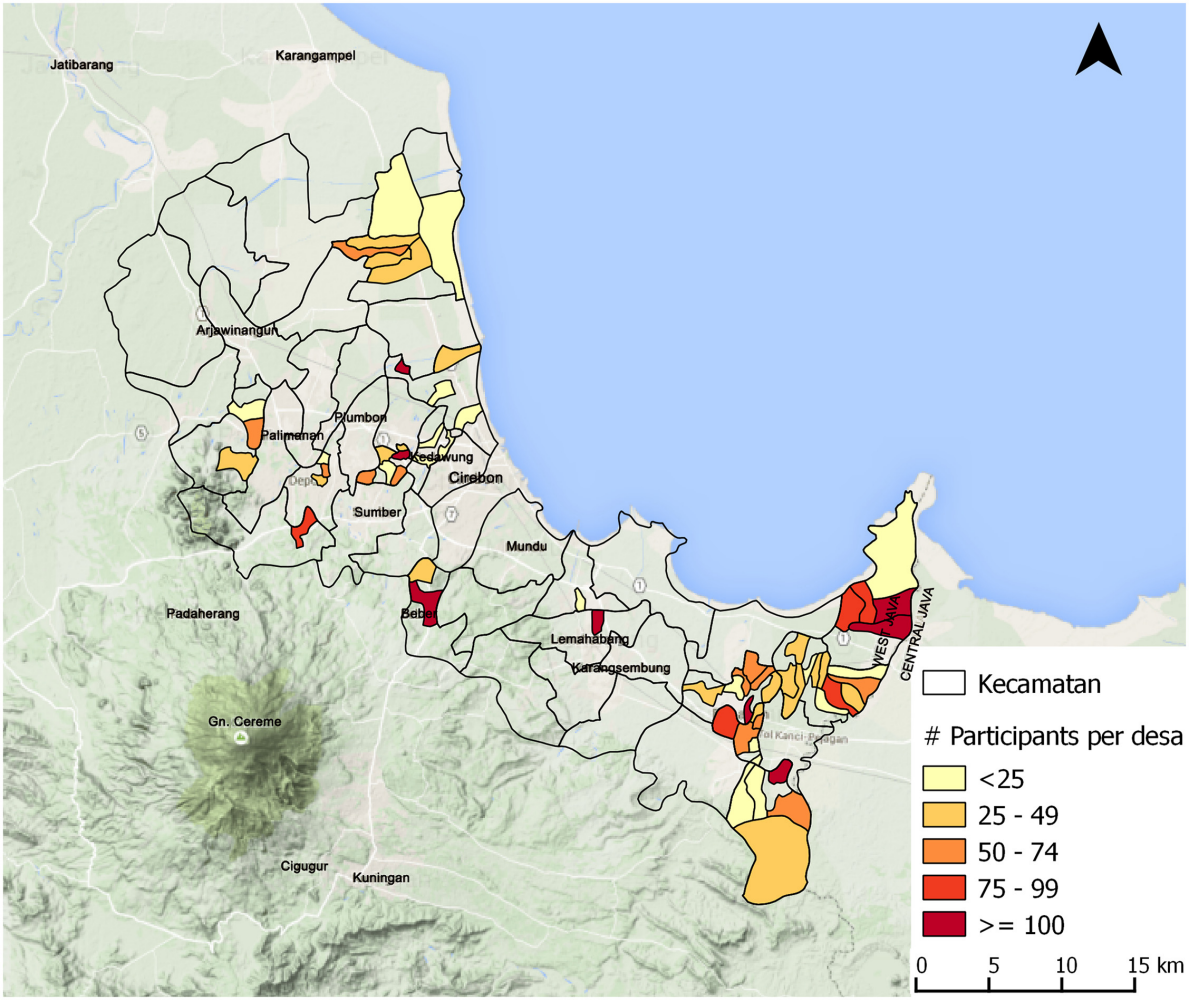
Number of participants contact events per village in Cirebon District.

In a 14 month time-span the SARI project organised 91 contact events in 62 villages, which were located in 16 different sub-districts (see Fig 1). More than 4,400 community members attended a contact event (see Fig 2), including 803 key persons (18%). On average 49 community members attended one event. Contact events were most frequently held at the village hall, family house and school. Testimonies were provided in 55% of the contact events. See Table 4 for more detailed information on the contact events. Providing a testimony was both difficult and rewarding for the participants affected by leprosy. Sometimes they attended a contact event a few times without giving a testimony before they felt confident and ready to do so themselves. Two responses:

*I felt very emotional when I told them about my experience with leprosy*. *Maybe because it is the first time I ever shared my feelings*. *I am very happy that I can let it go*. *I am calm and happy that the audience can appreciate this event (Event B44)*

*I enjoyed this event*, *I even joked around with my current condition of being still single*, *I never thought the audience would respond like they did and prayed for me to have a spouse (Event B13)*

**Table 4:** General overview about the contact events, audience and location.

Aspects	Descriptive	
Number of contact events	Count	91
Number of contact events per sub-district	Mean	5.7
	Range	1–12
Number of contact events per village	Mean	1.4
	Range	1–5
Number of participants	Count	4443
Students	Count (%)	1978 (44)
General public		1689 (38)
Key persons		803 (18)
Number of participants per contact event	Mean	48.8
	Median	28
	Range	2–603
Key persons		
RW/RT	Count (%)	280 (34.9)
Village official		232 (28.9)
PKK*		109 (13.6)
Religious leaders		108 (13.4)
Village leader		34 (4.2)
Teacher		21 (2.6)
Others**		27 (2.4)
Location		
Village hall	Count (%)	40 (44)
House		24 (26.4)
School		18 (19.8)
Mosque		8 (8.8)
Community Health Care Centre		1 (1.1)
Testimony given		
Yes	Count (%)	55 (60)
No		36 (40)

* PKK = Pembinaan Kesejahteraan Keluarga = Members of a women’s organisation

** Others = Military and policemen

### Impact of the contact intervention

Members in the audience: i) were actively involved (e.g. made notes, asked questions, shared personal experiences and gave an unplanned testimony), ii) were emotionally touched by the testimonies, iii) asked for or motivated others to get a health check-up due to a concerns being affected by leprosy, iv) requested more comics to distribute in the community, v) asked to start a participatory video process in their own sub-district, and finally vi) requested more contact events. There were also challenges. These related to convincing key persons (e.g. head of village) of the value of a contact event, practical/logistical challenges (e.g. weak audio system, inappropriate venue, too many people and limited time), the audience (e.g. tired, less involved) or to the SARI team (e.g. cancelations, delayed). In this section of the results, the focus lies on the impact of the contact events on knowledge, attitudes and the coverage.

#### Knowledge

Before the contact event, 87.2% perceived that leprosy can be cured and 33.1% thought that leprosy can be contagious also after taking medicine. These numbers increased to 98.3% and reduced to 7.4%, respectively, right after the event and even more important remained stable also more than three months after the contact event (see Table 5).

**Table 5:** Results 6-QQ: Knowledge questions pre, post (n = 769) and post-3-month (n = 114).

	% Yes pre	% Yes post	% Yes post-3-month
Q1. Can leprosy be cured?	87.2	98.3	98.2
Q6. Is leprosy contagious after taking medicine?	33.1	7.4	7

Of the 58 respondents in the end-survey who attended a contact event between 3 and 20 months earlier, 52 (89.7%) indicated that they still remembered information provided during the event. When asked what they remembered, the most frequent answer was “leprosy is not contagious if medication is taken” (n = 15), followed by “leprosy can be cured” (n = 13). Several participants mentioned that they remembered from the event to not stigmatize, discriminate or exile people affected by leprosy (n = 9) and to support them (n = 4). Table 6 provides an overview of the top 8 aspects mentioned most frequently by respondents.

**Table 6:** Top 8 aspects remembered by respondents end-survey who joined a contact event and remembered information (n = 52).

Aspects remembered	Count
1. Leprosy is not contagious if medication is taken	15
2. Leprosy can be cured	13
3. We should not stigmatize/ discriminate/ exile people affected by leprosy	9
4. There is medication at Community Health Care Centre	8
5. Symptoms of leprosy	7
6. Leprosy is not a genetic disease	5
7. We should support people affected	4
8. Medication is free	3

The participants of the FGDs remembered detailed and correct information about leprosy more than three months after the contact events. This information included the cause, symptoms of leprosy, types of leprosy, contagiousness, route of transmission, treatment, and effect of the medication on contagiousness. They also mentioned common misconceptions and corrected these. For instance, they said that leprosy is not genetic nor a curse from God and that leprosy cannot spread by shaking hands or eating food prepared by a person affected by leprosy. On a few occasions, misinformation was identified that could foster stigmatization. Three respondents in the final survey said that leprosy is not a contagious disease and one participant in a FGDs said that leprosy can be spread by mosquitoes.

Several interviewees indicated during the short informal interviews that took place after the contact events that this was the first time they received information about leprosy and that is was very useful. Two quotes:

*Oh this [contact event] is a must*! *If I am not mistaken*, *this is the first time we ever have counselling about leprosy (Event B31)*

*This [contact event] is very beneficial and useful*, *we were not very aware about leprosy*, *how to treat it*, *how to cure it*. *But through this event*, *we became very clear of it (Event B16)*

The increased knowledge influenced the feelings and attitudes of the interviewees towards persons affected with leprosy. They said that due to the new information about leprosy they were not worried anymore for infection and hence were willing to interact with people affected with leprosy as illustrated here:

*This [contact event] is very good*, *I understand more*. *I used to be very afraid to visit someone who have leprosy*, *but now I am not afraid (Event B26)*

#### Labelling, stereotyping, separation, status loss and discrimination

The EMIC-CSS and SDS total score means and 95% confidence intervals were calculated for the four different intervention areas. The largest differences between pre and post measurements occurred in the ‘Counselling—Contact’ and ‘Contact—SED’ areas (see Table 7). There was a difference in EMIC-CSS total score (2.66) also in the control area, in contrast to the SDS total score (0.31) that remained almost equal. This difference in EMIC-CSS score was nearly significant. The Smallest Detectable Changes of the EMIC-CSS and SDS with 15 items are respectively 0.81 and 0.61 [44]. The differences in EMIC-CSS and SDS scores in the intervention area are greater than these Smallest Detectable Change. The multiple linear regression revealed a significant difference in the areas where the contact intervention was implemented, in contrast to the ‘SED—Counselling’ and control areas. The effect sizes found in the different intervention areas are displayed in Table 8. Again, the largest effect sizes were found in the ‘Counselling—Contact’ and ‘Contact—SED’ area.

**Table 7:** Results univariate and multivariate analyses of total score EMIC-CSS (range 0–28) and SDS (0–21) pre and post by intervention area.

	Mean pre (95% CI)	Mean post (95% CI)	Mean difference (95% CI)	Un-adjusted *P* value	Adjusted mean difference (95% CI)	Adjusted *P* value
**EMIC-CSS**						
Counselling Contact*	14.3 (11.7–17.0)	10.9 (8.91–12.9)	3.43 (1.65–5.21)	<0.001	3.13 (1.57–4.70)	<0.001
Contact SED**	14.6 (13.6–15.6)	10.5 (6.35–14.7)	4.04 (1.76–6.32)	0.001	3.02 (1.02–5.02)	0.003
Counselling SED ***	14.1 (12.1–16.2)	12.0 (9.48–14.5)	2.14 (0.73–5.02)	0.142	1.61 (1.01–4.22)	0.227
Control****	12.9 (9.3–16.5)	9.90 (6.82–13.0)	2.97 (0.37–5.57)	0.026	2.66 (0.05–5.38)	0.054
**SDS**						
Counselling Contact*	9.33 (8.13–10.5)	7.28 (6.46–8.10)	2.05 (0.92–3.18)	<0.001	2.37 (1.23–3.50)	<0.001
Contact SED**	8.96 (7.84–10.1)	6.93 (4.76–9.10)	2.03 (0.47–3.59)	0.011	2.50 (0.90–4.10)	0.002
Counselling SED ***	9.38 (8.01–10.8)	7.80 (6.53–9.07)	1.58 (0.39–3.55)	0.115	1.58 (0.37–3.54)	0.111
Control****	8.55(7.72–9.38)	8.52 (6.94–10.1)	0.03 (-1.66–1.72)	0.973	0.31 (-2.25–1.63)	0.754

* n = 72+128,

** n = 52+126,

***n = 42+60,

****n = 47+61

**Table 8:** Effect size based on adjusted means EMIC-CSS and SDS.

	EMIC-CSS	SDS
Counselling Contact	0.47	0.54
Contact SED	0.46	0.57
SED Counselling	0.24	0.36
Control	0.40	0.07

The mean scores for the sub group of respondents of the final survey who had attended a contact event (n = 58) 3 to 20 months earlier were also calculated. We calculated the adjusted mean scores because of the differences in demographic pattern between the samples. The adjusted mean score of the EMIC-CSS in this group was 8.63, a reduction of 4.95 compared to the mean score at baseline (p <0.001). For the SDS this was 6.94, a difference of 3.56 compared to mean score at baseline (p <0.001). The effect sizes for this sub-group were 0.75 for EMIC-CSS and 0.81 for the SDS, respectively.

**Fig 3:**
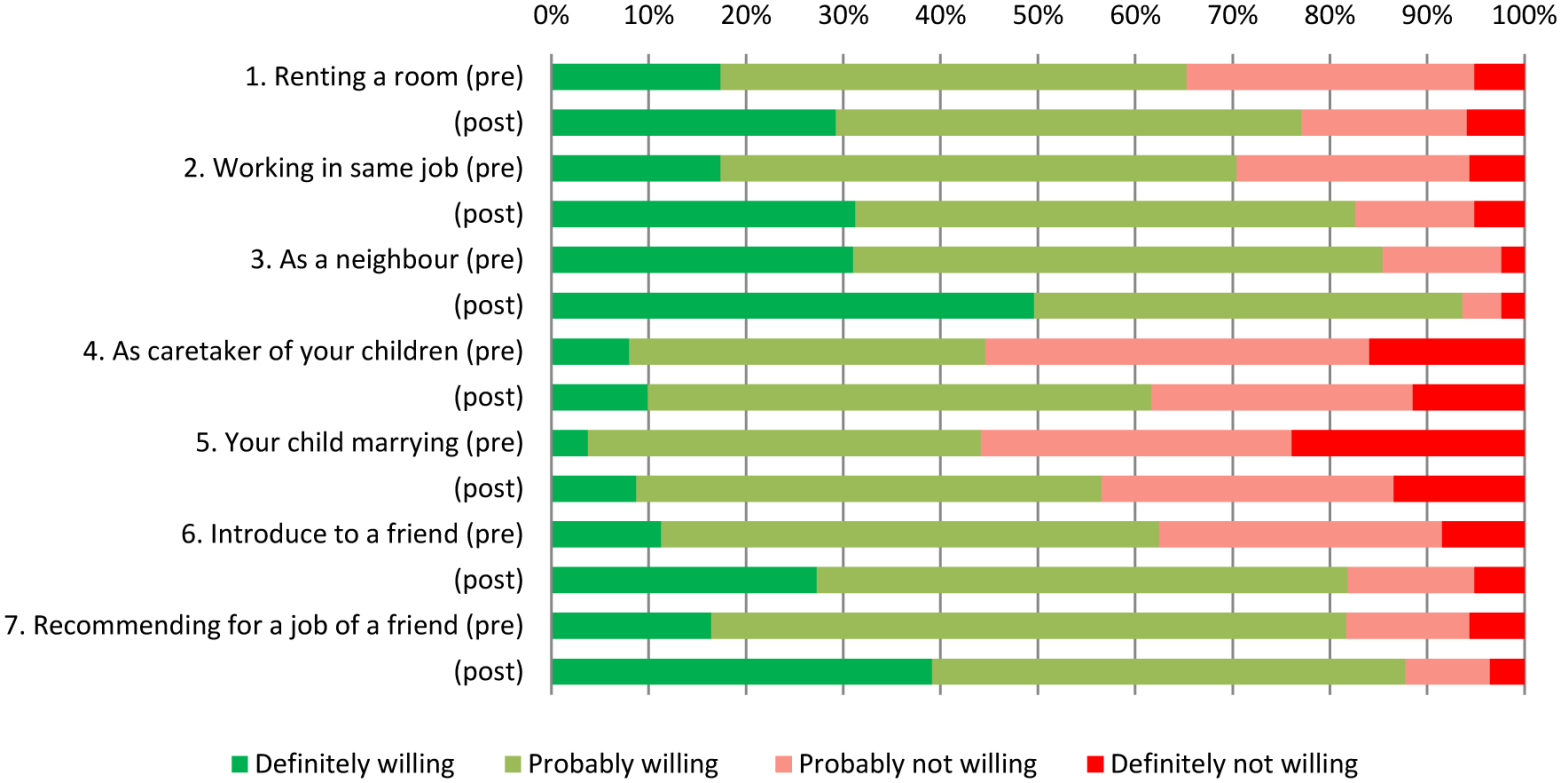
SDS items pre (n = 213) and post contact intervention area (n = 253).

For the SDS, the individual items were explored in more detail. Fig 3 depicts the distribution of answers at baseline and compares these with the answers post intervention in the contact intervention areas. The largest differences in terms of willingness (‘definitely willing’) were found with regard to having someone affected by leprosy as a neighbour or recommending him or her for a job. The smallest differences occurred in having someone affected by leprosy as a caretaker of your children or having your child marry someone affected by leprosy.

In the 6-QQ results we also see large differences in recorded attitude. However, after three months, these positive changes had reduced somewhat as illustrated in Table 9.

**Table 9:** Results 6-QQ: Attitude questions pre, post (n = 769) and post-3-month (n = 114).

	% Yes pre	% Yes post	% Yes post-3-month
Q2. 'Do you feel comfortable when you meet people who have leprosy?'	17.7	62.8	40.7
Q3. 'Are you worried for contagion of you shake hands with a person who has leprosy?'	79.7	31.1	57
Q4. 'Are you worried if you eat food prepared by a person who has leprosy?'	88	37	65.5
Q5. 'Are you worried if a person affected by leprosy marries with a family member?'	86.8	39.2	65.8

During the FGDs, both the village leaders and the women mentioned they were less afraid for persons affected with leprosy due to the contact event, they understood that persons with leprosy do not need to be avoided and that they can shake hands without getting infected. However, one village leader said he washes his hands after shaking hands with someone who has leprosy and another village leader indicated that he was afraid for the persons who did not have medication yet. Two quotes:

*There is a change in our attitude*. *We are not afraid anymore that leprosy can be infectious*. *(FGD Women of religious gatherings)*

*I am not afraid anymore for persons affected with leprosy who already have medication*. *But I limit myself from interacting with those who have not started medication*, *I am afraid of getting infected (FGD Village leaders)*

One village leader indicated stigma can be reduced but that it is difficult to eradicate stigma completely. Another village leader talked about rights when the facilitator asked why it was important to reduce stigma:

*Because everybody has the right to live a happy life (FGD Village leaders)*

Contrary to what is mentioned above, the students indicated in the FGDs that they were more afraid of getting infected with leprosy after the contact event. One student indicated this is because they are now aware of the impact of leprosy and the possible impairments it could cause. Another change mentioned by some students was that they were more aware of the importance of keeping a healthy lifestyle to prevent themselves from getting infected with leprosy or any other disease. Though saying they were more afraid, this did not prevent them from intending to be more supportive and caring towards persons affected by leprosy. Several students said they will motivate persons affected with leprosy to get medication but also tell them to keep their hopes up and not to be stressed with their condition. Important to mention is that all students were talking about *if* they would meet someone affected with leprosy because none of the students had any contact with someone affected with leprosy.

*When we meet them*, *we should motivate them to keep their hopes high because leprosy can be cured*. *When they are down*, *we should motivate them (FGD Students)*

Expressing a desire for change—as shown in the preceding quote—does not necessarily mean that the participants will act on this in real life. Besides an increased willingness to interaction, actual behaviour changes were mentioned by village leaders and women. One village leader indicated that he invited someone with leprosy to his house. The women of the religious gatherings mentioned that they meet more frequently with persons affected with leprosy after the contact event.

### Coverage

During the informal interviews people in the audience stated the importance of and willingness to share this information with others.

*It is very useful*! *As the village chief*, *I can share this information to the community*. *They should not be afraid of leprosy*. *They should not excommunicate the patient*. *Leprosy is not infectious*. *If it were not for this event then I would not know anything about leprosy*. *(Event B27)*

Respondents of the final survey who attended a contact event were asked whether they had shared the information of the event with others and if so with how many others. Of the 58 respondents, 44 (75.9%) said they shared the information with others. On average, they shared this information with 10 others (mean 16.5, median 10, minimum 2 and maximum 99). In particular, religious leaders had shared the information to large groups during religious teaching (shared with ~25), during the praying for a person who passed away (with ~60) and while reading Quran (shared with ~100).

In the FGDs, the village leaders, women of the religious gatherings and students indicated they passed on the information about cause, symptoms and treatment to family, neighbours and friends. One woman said she had shared the information to students and instructed them to go to the HCs for medication if they have symptoms. Village leaders said they informed the public during religious gatherings and community meetings. In addition, village leaders and women of the religious gatherings also provided information about medication and the importance of visiting the HCs to the persons affected with leprosy.

## Discussion

In this paper we showed that a contact intervention with education, testimonies, comics and participatory videos was highly effective in increasing knowledge about leprosy and reducing leprosy-related stigma in Cirebon District, Indonesia. To our knowledge, the SARI project is the first study that assessed the effect of a contact intervention in the field of NTDs. Notable aspects of this study are the cluster-randomized, controlled intervention study design, using mixed-method, the preceding exploratory study (to identify local beliefs and misconceptions), the testing in actual communities (rather than small samples in selected settings), participation at all levels of persons with disabilities and engagement of people affected by leprosy as agents of change.

The contact intervention was highly effective for community members who attended the contact events (large ES) and moderately effective for community members (moderate ES) living in the villages where contact events were organised. For both groups, these differences were significant. The findings obtained during the implementation of the interventions and in the final survey showed a positive effect on both knowledge and reported attitudes. For example, the audience remembered detailed information about the disease leprosy. But also respondents’ willingness (‘definitely willing’) of having someone affected by leprosy as a neighbour or recommending him or her for a job increased by about 20%. In the field of leprosy there is a lot of experience with education, but not with contact as an intervention. The effect of education alone is subject of discussion [14]. Some studies found positive effects of education on reducing leprosy-related stigma [48,49], but in general, the effects are seriously questioned [32,50,51]. In other fields than leprosy, such as HIV/AIDS and mental health, studies have assessed the effect of contact interventions (with videos, lectures) and many found an association between ‘contact’ (sometimes combined with education as was also the case in this study) and improved attitudes and reduced social distance [21,23,26,52–57].

The findings of the baseline revealed contrasting attitudes towards leprosy. We found a lack of knowledge in the community about the disease leprosy, particularly regarding the aetiology and way of transmission. The baseline also exposed processes of labelling, stereotyping, separation, status loss and discrimination against people affected by leprosy. The combination of a lack of knowledge and stigmatizing attitudes towards people affected by leprosy was found elsewhere, for example, in Brazil, Paraguay, Nepal and India [13,58–60]. On the other hand, we were positively surprised by the contrasting stories of care and support and the acknowledgements of human rights by community members at baseline. We are not aware of studies that focus on positive attitudes towards leprosy in the community and we suggest more attention to these experiences.

The results presented in this paper are very promising, but some critical issues need to be addressed. First, will the effect of the intervention remain and strengthen itself or will it erode with time? In this study, the final survey with the EMIC-CSS and the SDS, FGDs and 6-QQ took place between 3–20 months after the contact events. Compared to other studies this is a relatively long follow-up period. With one method (6-QQ) we collected data right after the events and after more than three months which allows us to give an indication of the effect over time. We found that the positive impact on knowledge gained during the events remained, but that the positive changes in attitudes reduced after 3 months. In the field of mental health, several studies that assessed the effectiveness of a contact intervention found that the effect reduced at follow-up periods ranging from one to six months [55,56,61]. What exactly happens over time and if the effect can be maintained through reminders (in this study comics were distributed that could serve as reminder of the event) or repetition are important questions that should be addressed in future research.

Second, is this intervention sufficiently replicable and can it be scaled up? We aimed to design a low-cost and easy-to-replicate intervention because of the resource-poor settings in which leprosy is most prevalent. The development of the indirect contact material (videos and comics) and the organisation of the contact events do not require specialised knowledge, but a certain level of understanding and skills of the facilitators or implementers was crucial in the SARI project. The intervention does not require expensive technology, but some costs and time are involved. Therefore, the replication will need to be supported by the leprosy programme or by non-governmental organizations or disabled people’s organizations. Future research should focus on enhancing scalability. Wong wrote: “while outcome evaluation assesses the effectiveness of a programme, process evaluation will explain why a programme failed or succeeded in reducing stigma” [42]. The ‘why’ question, but also how the approach can it be better combined with existing activities and services are important questions to enhance scalability in the future.

Third, why was there a near-significant difference in EMIC-CSS total score in the control area? The EMIC-CSS represents the perception of respondents regarding the attitudes and practice of others in their community concerning leprosy and leprosy-affected persons. This difference indicates that respondents in the control area perceive that aspects of leprosy-related stigma (e.g. shame, wish to conceal, difficulties in getting married) have improved in the community. Interestingly, the SDS score, representing their own personal attitudes, did not change in the control group. If there had been a genuine change in stigma level in the community due to other factors than the intervention, it is likely that the SDS scores would have changed also, as they did in the other interventions areas. It is very unlikely that the observed difference in results between the two instruments was due to poor validity of either one. A rigorous validation study showed both instruments to have good cultural validity in the Cirebon context [44]. We believe it is more likely that people in the control area may have been aware of the recent efforts to reduce stigma (the purpose of the SARI project was explained as part of the informed consent procedure) and may have expected these efforts to have reduced leprosy-related stigma in the community. However, when their own attitudes were tested, they had not changed. A similar issue occurred in a study of Angermeyer et al. in Germany [62], where a difference was found between the perceived public opinion and personal attitudes towards people who are mentally ill. It was hypothesised that people may have been aware of numerous anti-stigma efforts. Angermeyer et al. stated “these initiatives and programs may have had stronger effects on stigma perception than on actual stigma”. We believe that this phenomenon is inherent in and a disadvantage of measuring perceived attitudes and practices in this way.

Fourth, did the behaviour of community members change? The need for a translation of changes in attitudes to actual behaviour change is very important and has been stressed by others also [15,32]. The few examples of actual behaviour change mentioned in the FGDs in our study are promising, but do not allow firm conclusions that behaviour has also changed. It would be valuable to focus more on actual behaviour in the future, for instance through observations and by assessing the experiences of people affected.

Our findings have to be considered in the context of the study’s limitations. First, the randomization and selection did not work as well as designed and hence additional analyses were needed to calculate the adjusted effects. We were not able to adjust our multivariate analyses for clustering, most likely due to the number of clusters and the sometimes limited observations per cluster. Hence, the confidence intervals presented are slightly narrower than would have been the case if adjustment would have been possible. We believe that this would not have changed our overall conclusions. Second, the findings of the 6-QQ, informal interviews and FGDs may have been influenced by selection bias and the possibility of respondents giving socially desirable answers. Though measures to prevent this were taken, the findings may be slightly more positive than the reality. Third, we assessed reported change in attitudes and behaviour, which may be different from actual behaviour, as mentioned above. Fourth, for interpretability of the SDS and EMIC-CSS scores, the differences should ideally be compared with the minimally important changes (MIC). However, the size of the MIC calculated using an anchor method [63] are not yet available [44].

### Conclusion

The contact intervention implemented in this study was effective in increasing knowledge and improving reported attitudes in Cirebon District, Indonesia. The contact intervention is relatively easy to replicate elsewhere and does not require expensive technology, but more research is needed to improve scalability. Video and written materials that can guide practitioners on how they could design, implement and assess a contact intervention will follow. The material will be made available on Infolep, the international knowledge centre for access to information resources on leprosy and related subjects (see http://www.leprosy-information.org/keytopic/sari-project). Findings of this study support evaluation of a contact intervention to reduce stigma against other neglected tropical diseases and conditions.
